# Supplementary material for: Chromothripsis during telomere crisis is independent of NHEJ, and consistent with a replicative origin
Source: Genome Res. 2019 May;29(5):737–49. doi: 10.1101/gr.240705.118 (PMC6499312; doi:10.1101/gr.240705.118)
Supplement: Supplemental Material [file supp_gr.240705.118_Supplemental_file_1.zip › contigs/annotated_contigs/DB78/contig.2.DB78_length_323_mean_cov_12.5108359133.docx]

**DB78_length_323_mean_cov_12.5108359133**

```
ATCATATCATATGCTGTGGGCTTAGATTTGGCTTGTCATTTCTAAGACTCTCCTGGGAAGTTGTACGTAAAATAGTTTCTACTTAAAGT
 >chr13:77007599-77007720 - E=2e-58 p=3e-03
TAGAAGGAAATTTTTTTTTTTTGT|GTTTAAGT|GTGTAGTAACTTTTCTCTCAAGTTTAAAATACACCTTTCTTTATCTTCTCTTGGT
                         >chr13:76997557-76997749 - E=1e-88
TTTCTTTTTTTTTTCTTTGAGACGGATTCTTGCCCAGTCGCCCAGGCTGAAGTGCAATGGCGCGATCTCAGCTCACTGCAACCTCCGCC

TTCCGGTTTCAAGTGATTCTCCTGACTCAGCCTCC|TGAGTAGCTGGGATTACAGGCAT
```
